# Comparative analysis reveals a role for TGF-β in shaping the residency-related transcriptional signature in tissue-resident memory CD8^+^ T cells

**DOI:** 10.1371/journal.pone.0210495

**Published:** 2019-02-11

**Authors:** Artika P. Nath, Asolina Braun, Scott C. Ritchie, Francis R. Carbone, Laura K. Mackay, Thomas Gebhardt, Michael Inouye

**Affiliations:** 1 Department of Microbiology and Immunology, The University of Melbourne and The Peter Doherty Institute for Infection and Immunity, Melbourne, Victoria, Australia; 2 Cambridge Baker Systems Genomics Initiative, Baker Heart and Diabetes Institute, Melbourne, Victoria, Australia; 3 Cambridge Baker Systems Genomics Initiative, Department of Public Health and Primary Care, University of Cambridge, Cambridge, United Kingdom; 4 Department of Biochemistry and Molecular Biology, Monash University, Clayton, Victoria, Australia; 5 Department of Clinical Pathology and School of BioSciences, University of Melbourne, Melbourne, Victoria, Australia; 6 The Alan Turing Institute, London, United Kingdom; CCAC, UNITED STATES

## Abstract

Tissue-resident CD8^+^ memory T (TRM) cells are immune cells that permanently reside at tissue sites where they play an important role in providing rapid protection against reinfection. They are not only phenotypically and functionally distinct from their circulating memory counterparts, but also exhibit a unique transcriptional profile. To date, the local tissue signals required for their development and long-term residency are not well understood. So far, the best-characterised tissue-derived signal is transforming growth factor-β (TGF-β), which has been shown to promote the development of these cells within tissues. In this study, we aimed to determine to what extent the transcriptional signatures of TRM cells from multiple tissues reflects TGF-β imprinting. We activated murine CD8^+^ T cells, stimulated them *in vitro* by TGF-β, and profiled their transcriptomes using RNA-seq. Upon comparison, we identified a TGF-β-induced signature of differentially expressed genes between TGF-β-stimulated and -unstimulated cells. Next, we linked this *in vitro* TGF-β-induced signature to a previously identified *in vivo* TRM-specific gene set and found considerable (>50%) overlap between the two gene sets, thus showing that a substantial part of the TRM signature can be attributed to TGF-β signalling. Finally, gene set enrichment analysis further revealed that the altered gene signature following TGF-β exposure reflected transcriptional signatures found in TRM cells from both epithelial and non-epithelial tissues. In summary, these findings show that TGF-β has a broad footprint in establishing the residency-specific transcriptional profile of TRM cells, which is detectable in TRM cells from diverse tissues. They further suggest that constitutive TGF-β signaling might be involved for their long-term persistence at tissue sites.

## Introduction

TRM cells are a recently identified subset of memory T cells that reside in peripheral tissues without re-entering circulation [1–4]. TRM cells have been identified in a number of barrier and neuronal tissues including the skin, lung, gut, liver, female reproductive tract, and brain, where they have been shown to offer superior protection against local re-infection compared to their circulating central (TCM) and effector memory (TEM) CD8^+^ T cell counterparts [2,3,5–9]. TRM cells that localise to the epithelial and neuronal tissues commonly express the cell surface molecule CD103 (integrin αE), which is thought to promote TRM persistence through adhesive interactions with epithelial cell-expressed E-cadherin [3,10–14]. However, the local tissue-derived signals that instruct and control the development and persistence of TRM cells at tissue sites are not completely understood. Understanding the mechanisms underlying these processes, which provide rapid and enhanced site-specific immunity, have the potential to enable rational vaccine design.

The role of cytokines in the differentiation and maintenance of circulating memory T cell subsets is well documented [15,16], and there are established links between local tissue-derived cytokines and tissue residency [8,11,12,17–19]. In particular, TGF-β activity is critical for the development of CD8^+^ CD103^+^ TRM cells in the skin, gut and lungs, although TGF-β -independent TRM cells have been described during protracted bacterial infection in intestinal mucosa [8,11,12,17,18,20]. For example, studies have shown that TRM cells with defective TGF-β receptors, which are unable to respond to TGF-β signals, do not up-regulate CD103 expression and are incapable of maintaining residency at tissue sites [8,11,12,17,18].

It has recently been shown that mouse CD8^+^ CD103^+^ TRM cells isolated from skin, gut, and lung share a TRM-related transcriptional program, suggesting a common molecular machinery underlying their development, maintenance, and possibly function in peripheral tissues [12]. However, the role of TGF-β in shaping the TRM cell transcriptome, in particular, the shared TRM-related gene signature has not been elucidated. In this study, we sought to determine to what degree the previously identified common, tissue-independent TRM-related gene profile [12], hereafter referred to as “TRM-related signature”, can be attributed to TGF-β signalling. To do so, we used RNA-sequencing to profile the transcriptome of murine CD8^+^ T cells stimulated *in vitro* by TGF-β. First, to identify a TGF-β specific gene signature, we compared the transcriptome of *in vitro* TGF-β-stimulated activated CD8^+^ T cells to unstimulated cells. We then compared this *in vitro* TGF-β-induced transcriptional signature to the TRM-related signature *in vivo* and found a substantial overlap in their transcriptional profiles, thus providing new insights into the central role of TGF-β signalling in shaping the transcriptional program of TRM cells from both barrier and non-barrier tissues.

## Methods

### Mice

Female C57BL/6 (wild-type [WT] B6) and gBT-I mice on C57BL/6 background, between the ages of 8 and 15 weeks, were used in this study and were bred and maintained under specific pathogen-free conditions in the Department of Microbiology and Immunology, University of Melbourne. The gBT-I mice express a transgenic T cell receptor that recognises the herpes simplex virus type 1 glycoprotein B (gB) peptide [21]. All animal experiments were approved by The University of Melbourne Animal Ethics Committee.

### Flow cytometry analysis

The suspension of gBT-I cells were stained for 15 min at 4ºC with the following fluorescence-conjugated antibodies for flow cytometry: anti-CD45.1 (A20), anti-vα2 (B20.1), anti-CD8α (53–6.7), anti-CD103 (2E7), all obtained from BD Pharmingen or eBioscience. Dead cells were excluded using propidium iodide or DAPI staining. Cells were analysed using the BD FACSAria flow cytometer at the Melbourne Cytometry ImmunoID Flow Cytometry facility (The University of Melbourne). Flow cytometry data were analysed using the FlowJo software (TreeStar).

### *In vitro* cell culture and RNA extraction

The *in vitro* culture system utlised in this study has been described previously [3,22]. Briefly, one gBT-I and one B6 spleen was harvested and processed into single cell suspensions by passing them through a 70 μm mesh. The B6 cells were coated with gB peptide (100 μg/ml) in 4ml RP-10 (complete RPMI-1640 with 10% fetal calf serum) per spleen at 37°C for 15 minutes, washed and taken up in RP-10 with 2 mg/ml of lipopolysaccharide (L4391, Sigma). 5ml of B6 splenocyte suspension was added to gBT-I splenocytes from ½ spleen in 35 ml RP-10 per T75 flask. On day 2, 3, and 4 the cultured cells were expanded with 500 U/ml of recombinant human interleukin-2 (IL-2). On day 5, the activated gBT-I cells were seeded at 12 million/5ml RP-10 and stimulated with or without 3 ng/ml of TGF-β for 40h in four conditions: Unstimulated, TGF-β, IL-2 or IL-2/TGF-β. The experiment was repeated three times, for three independent biological replicates. A total of 12 samples were prepared.

Total RNA was extracted from each sample by adding 200μl chloroform per 1mL TRIzol directly to cells, vortexing briefly, and incubating at room temperature for 5 minutes. The samples were centrifuged at 12,000g for 20 minutes at 4°C. The colourless upper aqueous layer was transferred to a new tube containing 500μl of Propan-2-ol, kept at room temperature for 10 minutes, and then centrifuged at 12,000g for 20 minutes. The supernatant was removed, RNA pellet was washed with 1ml of 75% ethanol, and the samples were centrifuged at 7,500g for 5 minutes at 4°C. Following the removal of the supernatant, the RNA pellet was air dried no longer than 5 minutes, and then resuspended in 20μl of sodium citrate dissolved in RNase-free water. DNA digestion with DNase-I was carried out with the RNeasy MinElute Cleanup Kit (Qiagen, CA).

### DNA library construction, RNA sequencing, and data pre-processing

Library preparation and sequencing were both performed by the Australian Genome Research Facility (AGRF; Melbourne, Australia). All 12 RNA samples were processed with the Illumina TruSeq Stranded mRNA sample prep protocol to make cDNA libraries. The resulting normalised and pooled libraries were clustered on the Illumina cBot cluster amplification system using the HiSeq PE Cluster Kit v4 reagents followed by sequencing on the Illumina HiSeq 2500 system with the HiSeq SBS Kit v4 reagents. Base calling and quality scoring were done with the standard Illumina pipeline, Real-Time Analysis (RTA) version 1.18.64 software. De-multiplexed raw FASTQ files containing 100bp PE reads were generated using Illumina’s bcl2fastq version 1.8.4 pipeline. The qualities of the raw sequence reads were assessed using FastQC version 0.11.3 [23]. Based on the quality reports, read trimming was not required (Phred score > 35).

### RNA-seq analysis

The 100bp paired-end reads were aligned to the mouse (mm10) reference genome using Tophat2 version 2.1.1 [24]. All mappings were performed with the default options, except the mate pair inner distance and standard deviation parameters, which were set to 0 and 65, respectively. The alignment for each biological replicate was performed independently, and only reads that mapped uniquely as pairs were retained for downstream analysis. Transcript assembly and quantification was carried out with Cufflinks2 version 2.2.1 [25]. Gene-level abundance was expressed as fragments per kilobase of exons per million fragments mapped (FPKM) values. An FPKM > 0.3 cut-off was applied to remove lowly expressed genes. Differential gene expression analysis was performed using Cuffdiff2 version 2.2.1 [26], which employs a beta negative binomial distribution model to capture fragment count uncertainty and overdispersion (variability across biological replicates), and uses a sampling-based approach to statistically test for differences in gene abundance between groups. Three comparisons were made: (1) TGF-β *vs*. Untreated, (2) IL-2/TGF-β *vs*. Untreated, and (3) IL-2/TGF-β *vs*. IL-2. Genes with Benjamini and Hochberg [27] adjusted *P*-values < 0.05 were considered as significantly differentially expressed (DE) in each comparison. Genes DE with FPKM values higher or lower in the TGF-β-treated groups than those in the TGF-β-untreated groups were defined as “up-” and “down-regulated” genes, respectively.

### Functional enrichment analysis of differentially expressed genes

We first identified the overlapping DE genes in the three comparisons. To explore the biological processes in which the subset of common DE genes were involved, Gene Ontology (GO) enrichment analysis of genes up- and down-regulated in the TGF-β-treated groups was performed using GOrilla [28], with 23,997 annotated genes in the *Mus musculus* genome (UCSC version mm10) provided as the background set. GO terms significant at FDR < 0.05 were then summarised into representative terms based on semantic similarity using REVIGO [29]. Summarisation analysis was performed using the RELSIM semantic similarity measure with a medium similarity cut-off (*C* = 0.7) on genes from *Mus musculus*.

### Enrichment analysis of TRM-related gene signature

To gain insight into the role of TGF-β in influencing the transcriptional signature of TRM cells, we tested whether a previously defined core TRM-related signature [12] was also differentially expressed in our three comparisons of TGF-β-stimulated to -unstimulated cells. This core signature comprises 37 genes that were identified as commonly DE in murine TRM cells from skin, gut, lung with respect to their circulating spleen (TEM and TCM cells) counterparts [12]. Since the genes in the TRM-related signature were profiled on a microarray-based platform (Affymetrix Mouse Gene 1.0ST arrays), only 35 core TRM-related signature genes common to both platforms (RNA-seq *vs*. microarray) were considered. These 35 genes were compared to the DE gene list obtained in each of three TGF-β-treated *vs*. TGF-β-untreated comparisons and the degree of overlap was determined. Only genes differentially expressed in the same direction in both studies were considered as overlapping. Bootstrapping was further performed to evaluate the statistical significance of the observed overlap. A total of 10,000 bootstraps were performed. For each bootstrap, the intersection between *k* and *m*_*i*_ genes randomly selected (with replacement) was calculated, where *k* was the number of genes in the TRM-related signature (N = 35) and *m*_*i*_ was the number of genes DE in comparison *i*. The enrichment *P*-value was calculated as the probability of an overlap being greater than or equal to the observed overlap.

Gene set enrichment analysis (GSEA) was further carried out to test the enrichment of 10 previously identified tissue-specific TRM transcriptional signatures (**Table A in S1 Table**) against the ranked list of genes DE between TGF-β-treated *vs*. TGF-β-untreated groups using the GSEA version 2.2.3 software downloaded from the Broad Institute website (http://www.broadinstitute.com/gsea/index.jsp) [30]. The predefined TRM cell-associated gene sets analysed included genes previously identified as significantly DE (|log2FC| > 1.5) in TRM cells isolated from skin, gut, lung, brain, and liver [12,31,32]. Briefly, the skin, gut, lung TRM associated gene sets comprised genes previously reported as either up- or down- regulated in murine CD103^+^ CD8^+^ T cells from skin, gut, or lungs after infection with HSV, lymphocytic choriomeningitis virus or influenza virus, respectively, when compared to their circulating HSV-specific TCM and TEM counterparts [12]. The brain TRM associated gene sets have been previously reported to be up- and down-regulated in mouse brain CD103^+^ CD8^+^ T cells relative to splenic CD103^−^ CD8^+^ T cells following vesicular stomatitis virus infection [31]. Likewise, genes previously identified as up- and down-regulated in murine liver TRM cells (CD69^+^ KLRG1^low^) *vs*. spleen TEM cells (CD69^–^ KLRG1^high^), following immunisation radiation-attenuated sporozoites (malaria vaccine), were used as liver TRM cell-associated gene sets [32]. The list of genes DE between HSV-specific TCM or TEM cells and naive T cells was used as a negative control gene set (**Table A in S1 Table**).

Enrichment analysis was performed on standardised, log2-transformed FPKM values for 10,941 expressed genes (FPKM > 0.3) across the 12 samples. First, GSEA ranked all the genes differentially expressed between the TGF-β-treated *vs*. TGF-β-untreated groups by expression fold change using the ‘Signal2Noise’ ranking metric, which scales the mean expression within each group by their respective standard deviation. This resulted in a list of genes sorted according to their association with TGF-β treatment, with the most up-regulated genes at the top end and the most down-regulated genes at the lower end. Next, the genes in the predefined gene set were tested for their overrepresentation at the top (or bottom) end of the ranked list. An Enrichment Score (ES) defined the degree of enrichment [30]. The enrichment *P*-values were computed by running 100,000 permutations of phenotype labels. Gene sets significant at the standard GSEA cut-off: a nominal *P*-value < 0.05 and FDR q-value < 0.25, were defined as significantly enriched.

## Results

### Experimental design and analysis of the RNA-seq data

**Fig 1:**
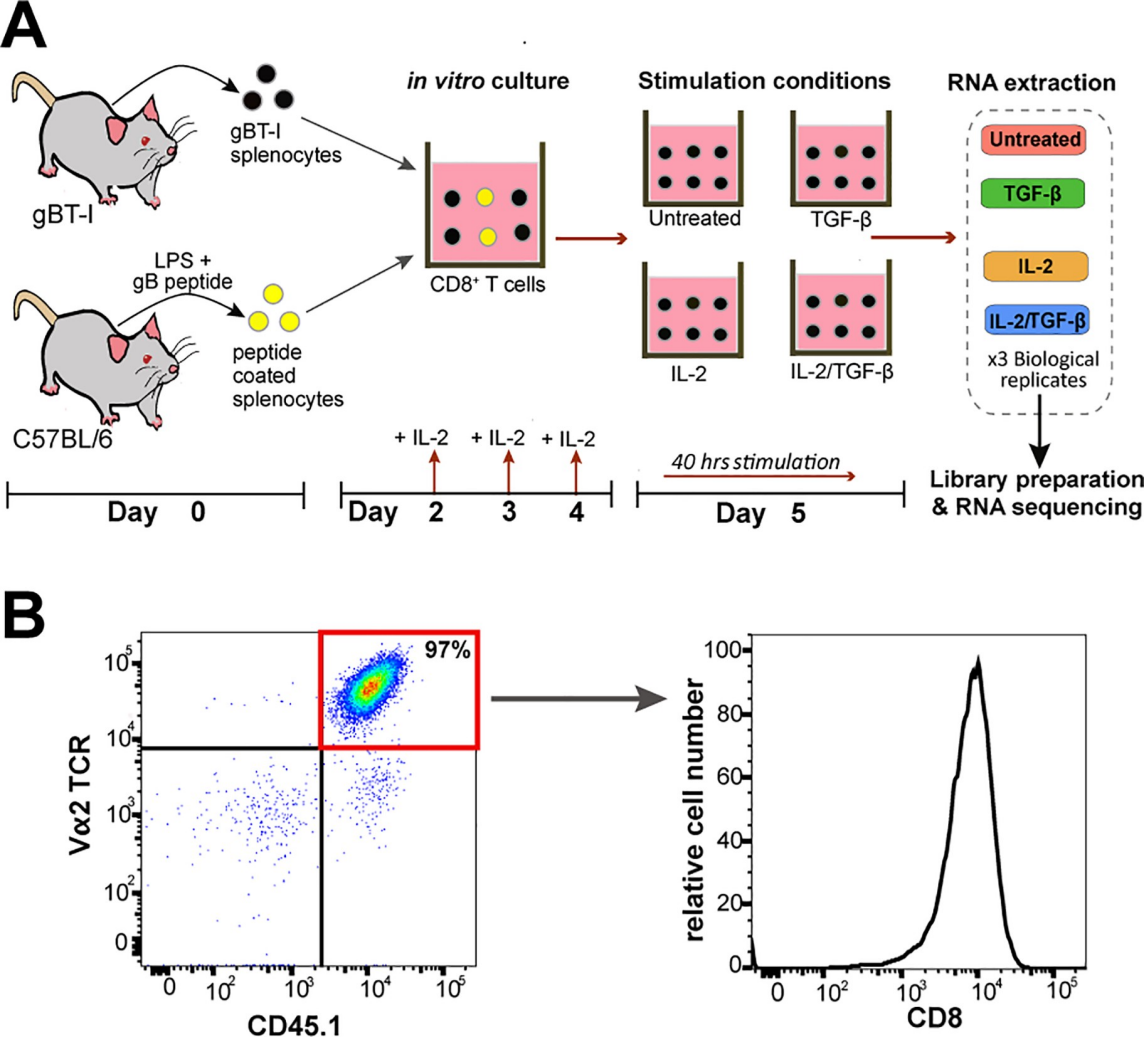
Schematic overview of the experimental design. (A) On day 0, wild type C57BL/6 splenocytes were coated with gB peptide and cocultured with gBT-I splenocytes in order to specifically activate gB-specific CD8^+^ T cells. Addition of IL-2 on days 2, 3, and 4 maintains T cell proliferation. At day 5, a highly pure population of activated CD8^+^ gBT-I T cells was subjected to four conditions: unstimulated control, TGF-β, IL-2, and TGF-β/IL-2. After 40h of TGF-β stimulation, cells were subjected to RNA extraction and RNA sequencing; N = 3 independent experiments. (B) Flow cytometric analysis of activated gBT-I cells on day 4. After pre-gating on live singlets, gBT-I cells are distinguished as CD45.1^+^, vα2 T cell receptor-positive (TCR^+^), and CD8^+^ cells. The representative dot plot (left) of CD45.1 and vα2 TCR staining, indicates the enrichment of gBT-I cells (red quadrant). The representative histogram (right) shows the expression of CD8 on gBT-I cells. The dot plot and histogram are representative of over 3 independent experiments.

As outlined in the experimental design workflow (**Fig 1A**), RNA samples were harvested from *in vitro* activated murine gBT-I cells that were stimulated with or without TGF-β in the presence or absence of IL-2. Prior to TGF-β stimulation, flow cytometry revealed the cell culture was highly enriched in activated CD8^+^ gBT-I T cell population (**Fig 1B**). During the incubation with TGF-β, the experiment included conditions with exogenous pro-survival cytokine IL-2 to mitigate any potential negative effects on cell survival *in vitro* within the 40h stimulation period. Three biological replicate samples were obtained for each TGF-β-stimulated (TGF-β; IL-2/TGF-β) and TGF-β-unstimulated (Untreated; IL-2) groups. A total of 12 RNA-seq libraries were prepared and sequenced on the Illumina HiSeq2500 platform at depths of 19.8–24.6 million 100 bp PE reads per sample (**Table B in S1 Table**). Read mapping, transcriptome assembly, gene-level quantification, and differential expression analysis were performed using the Tophat2/Cufflinks2/Cuffdiff2 pipeline as detailed in Methods (**S1 Fig**). Quality assessment of the raw reads with the FastQC tool [23] reported high quality reads with average quality (Phred) score greater than 35 for all the libraries. Across all libraries, paired-end read alignment rates to the mm10 mouse genome were high (range: 85.1–86.9% uniquely mapped reads) (**Table B in S1 Table)**.

### Global expression profiles were distinct between TGF-β-treated and TGF-β-untreated groups

**Fig 2:**
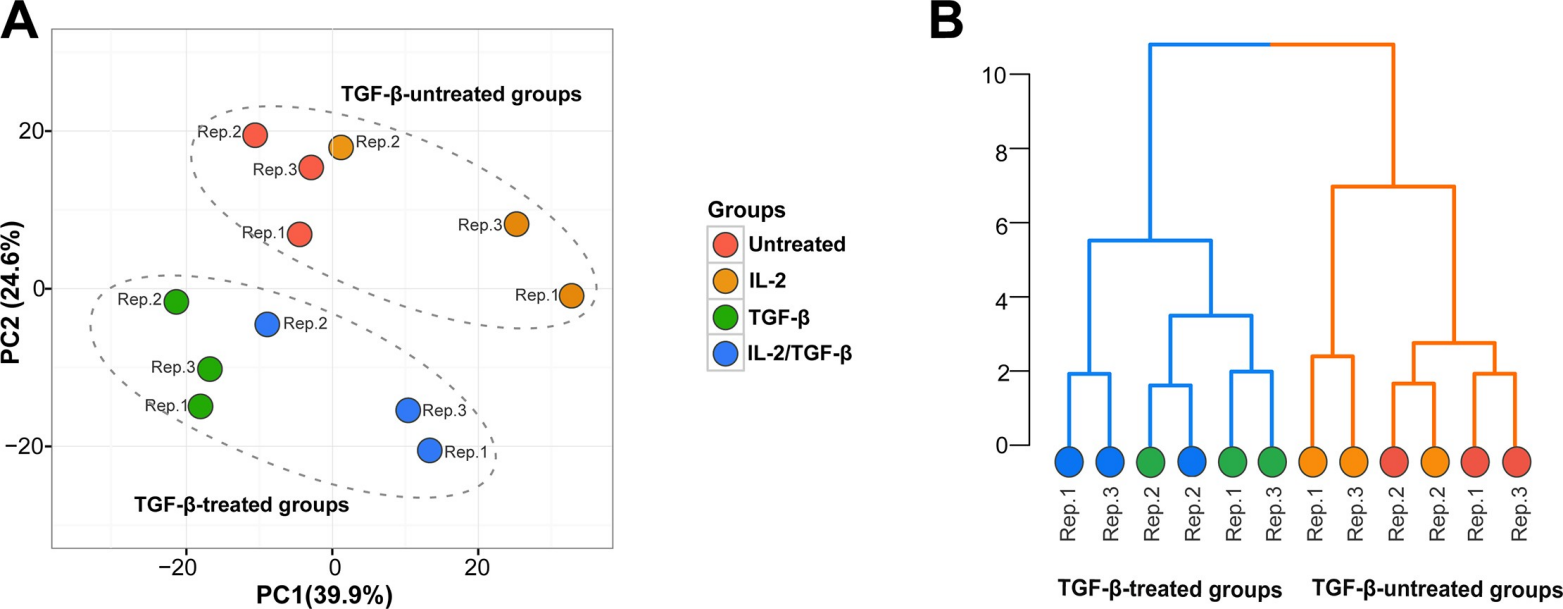
Principal component analysis and hierarchical clustering. (A) PCA plot (PC1 *vs*. PC2) of all 12 RNA samples. The numbers in parenthesis beside the PC labels denote the percentage of variance in the data explained by the respective PC. Clusters of TGF-β-treated (TGF-β and IL-2/TGF-Β) and TGF-β-untreated (Untreated and IL-2) groups, which separated along PC2, are circled. (B) Dendrogram from hierarchical cluster analysis of all the samples based on their expression profiles. Clustering was done using the Ward’s method with the “maximum” distances measure provided as the dissimilarity matrix. Dendrogram branches are coloured by TGF-β treatment: TGF-β-treated groups (blue) and TGF-β-untreated groups (orange). The dots represent biological samples coloured according to TGF-β treatment they received. Biological replicates (Rep.1-3) for each treatment have the same colour.

Exploratory analysis was performed on 10,941 abundant genes (FPKM > 0.3) across all samples. Principal components analysis (PCA) was carried out to characterise overall variation in gene expression profiles amongst TGF-β-treated and TGF-β-untreated samples. PCA showed that the first 3 principal components (PCs) explained more than 80% of total gene expression variance. PC1 (39.9%) was mildly related to IL-2 treatment (**Fig 2A**). The TGF-β-treated samples separated from the TGF-β-untreated groups primarily along the PC2 axis, which explained 24.6% of the variation in the data (**Fig 2A**). Hierarchical clustering showed two clear groups based on TGF-β treatment (**Fig 2B**).

### Identification of genes differentially expressed between TGF-β -treated and TGF-β-untreated groups

Differentially expressed genes between TGF-β-treated and TGF-β-untreated groups were identified using Cuffdiff2 [25] on three pairwise comparison groups: (1) TGF-β *vs*. Untreated; (2) IL-2/TGF-β *vs*. Untreated; and (3) IL-2/TGF-β *vs*. IL-2. The numbers of significantly DE genes obtained in each comparison are summarised in **Table 1**. The list of DE genes was further narrowed down based on fold change: greater than 2 –(|log2FC| > 1) and 4 –fold (|log2F| > 2) (**Table 1 and Table C in S1 Table**). Across all fold change cut-offs, in all the three comparisons, it was noted that TGF-β treatment resulted in mostly up-regulated genes. A similar trend was also seen with the top 30 most DE genes (based on |log2FC| > 2) common in all three comparisons (**S2 Fig**). Among this list, *Itgae* (CD103) was one of the top induced genes, providing transcriptional support to the role of TGF-β in up-regulating CD103 expression in TRM cells. The transcriptional upregulation of *Itgae* directly translated into higher levels of CD103 in TGF-β treated cells (**S3 Fig**). Hence, in this study, *in vitro* stimulation of CD8^+^ effector T cells with TGF-β produced a TRM-like phenotype.

**Table 1:** Differentially expressed genes identified by Cuffdiff2 in the TGF-β-treated vs TGF-β-untreated comparisons. The number of up- and down-regulated genes out of the total for each pairwise comparison across the different cut-off values.

Comparisons	FDR < 0.05	|log2FC| > 1 & FDR < 0.05	|log2FC| > 2 & FDR < 0.05
	Total (up-regulated / down-regulated genes)		
TGF-β *vs*. Untreated	849 (373 / 476)	240 (131 / 109)	57 (41 / 16)
IL2/TGF-β *vs*. Untreated	1261 (839 / 422)	448 (358 / 90)	93 (86 / 7)
IL2/TGF-β *vs*. IL-2	951 (436 / 515)	274 (162 / 112)	60 (52 / 8)
IL2/TGF-β *vs*. TGF-β	873 (725 / 148)	239 (225 / 14)	35 (33 / 2)

### Functional analysis of genes differentially expressed in TGF-β-treated groups

**Fig 3:**
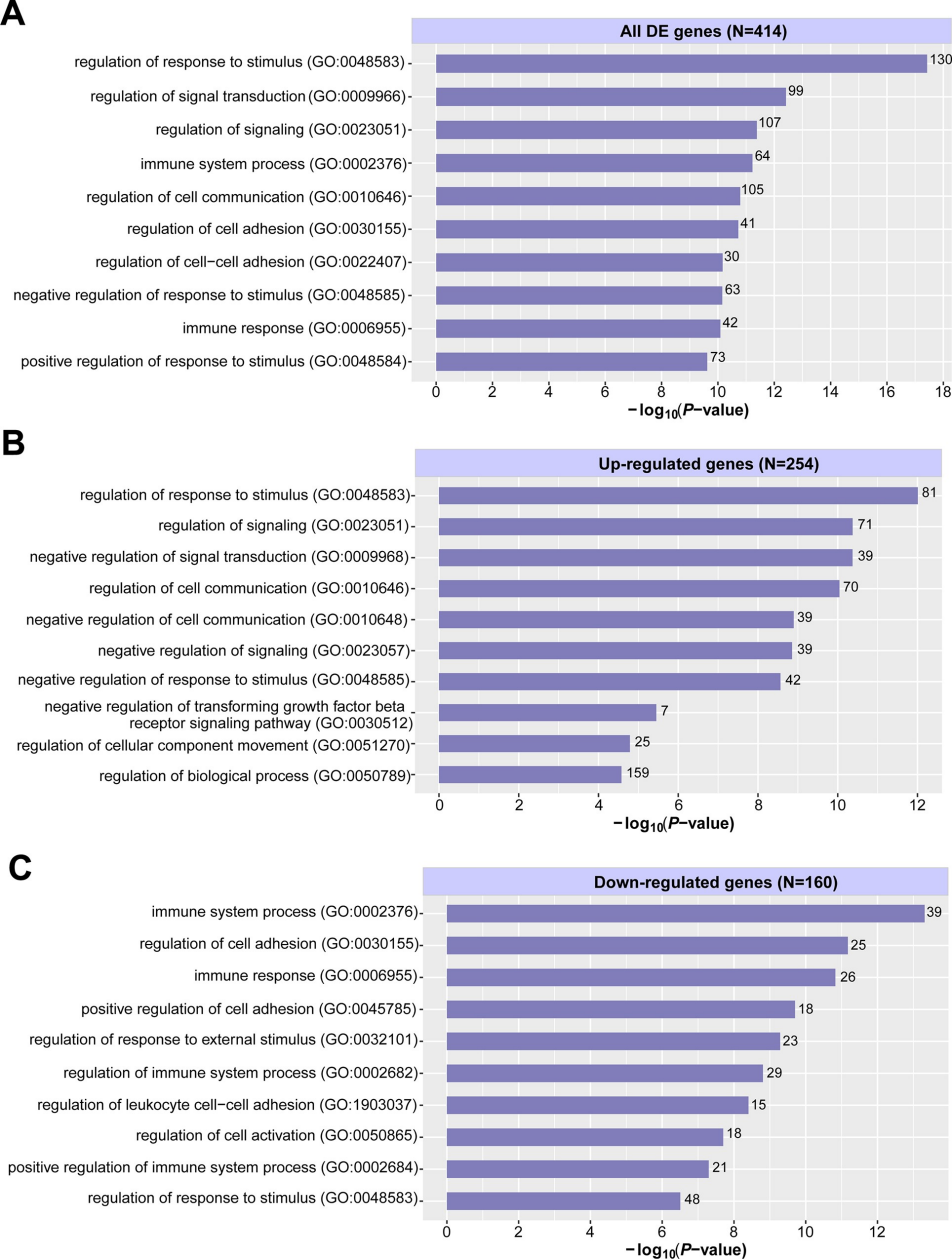
GO terms enriched among genes differentially expressed in the TGF-β-treated groups compared to their untreated counterparts. Top representative GO (biological processes) terms based on REVIGO output, enriched among (A) all the DE genes, (B) up-regulated genes, and (C) down-regulated genes in the TGF-β-treated groups. The GO terms (y-axes) were ranked according to their enrichment *P*-values (x-axes). The numbers on top of the bar plots in parentheses denote the total number of all DE, up-, and down-regulated genes with GO term annotations. The numbers at the end of each bar represent the actual number of all DE genes; up- or down-regulated genes that were classified to a particular biological process. All GO terms listed were significant at FDR < 0.05. All of the 254 common up-regulated genes in the TGF-β-treated groups were associated with GO terms. 160 out of 162 common down-regulated genes in the TGF-β-treated groups were annotated with GO terms.

To further characterise the genes differentially regulated in response to TGF-β stimulation, GO enrichment terms associated with biological processes were assigned to 416 common DE genes in the three comparison groups. GO enrichment was performed using GOrilla [28] on three sets of DE genes: up-regulated genes, down-regulated genes, and both sets combined. The significant GO terms (FDR < 0.05) were then further summarised into representative terms using REVIGO [29]. The top 10 over-represented GO terms among the DE genes in the TGF-β-treated groups, ranked by enriched *P*-values, are shown in **Fig 3**. All the 254 up-regulated genes were associated with at least one GO term. The most enriched biological processes among the up-regulated genes were related to the regulation of signalling, signal transduction, cell communication and cell movement (**Fig 3B**). 160 of 162 down-regulated genes had GO term annotations. These genes were largely involved in regulation of cell adhesion and response to stimulus (**Fig 3C**).

### Transcriptional profiles of TGF-β-treated T cells were enriched for TRM-related gene signature

**Fig 4:**
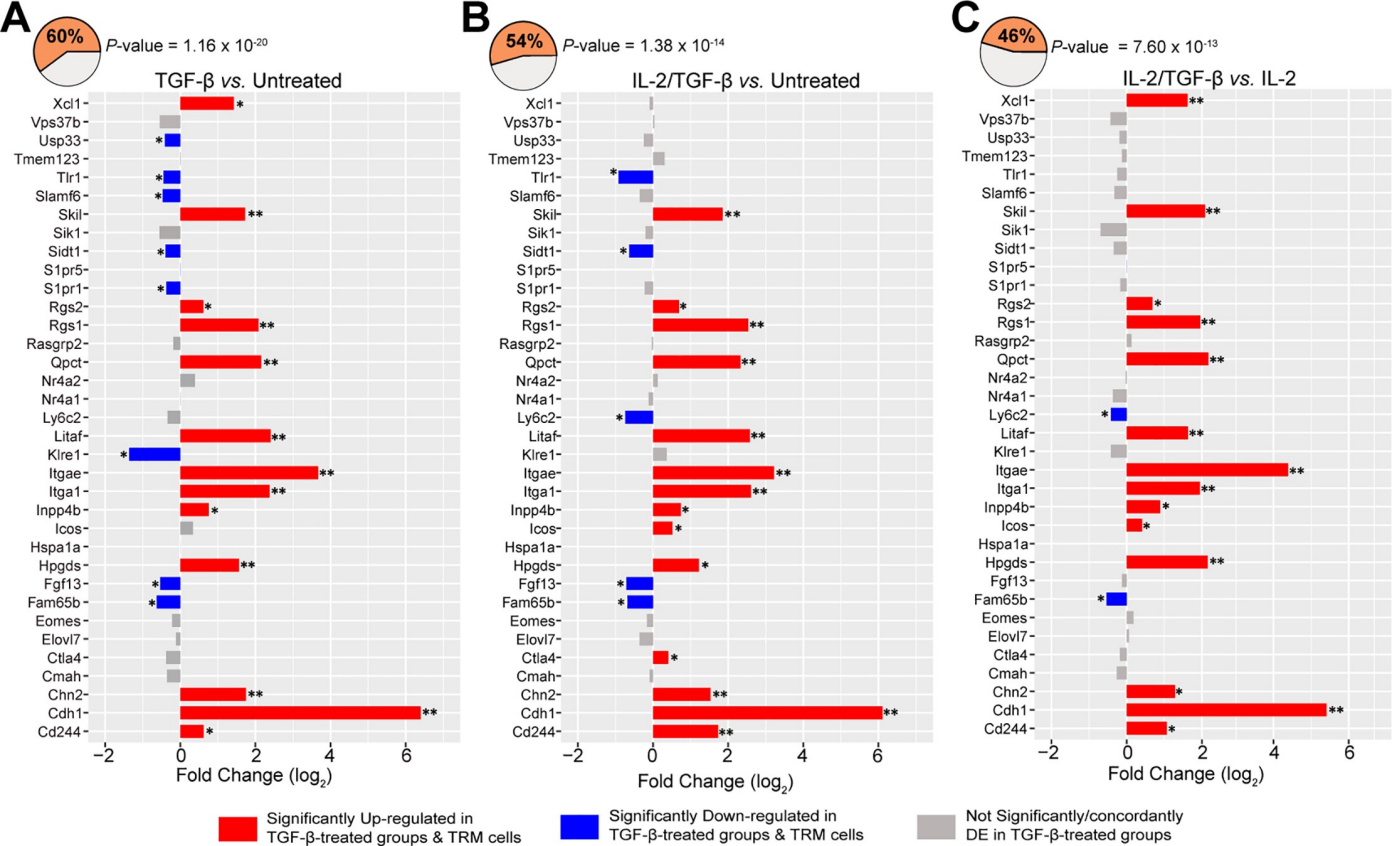
TGF-β induced transcriptional profiles are enriched for the TRM-related signature genes identified in murine TRM cells. (A-C) The bar plots show the log2 fold change in expression (x-axes) of the 35 TRM-related signature genes in each of the pairwise comparison of the TGF-β-treated groups *vs*. TGF-β-untreated groups. The bars are colour-coded based on their expression in both TRM cells and TGF-β-treated groups. Consistently up-regulated genes are depicted in red, and down-regulated genes are shown in blue. Grey represents genes that were either not significantly DE in TGF-β-treated groups *vs*. TGF-β-untreated groups or whose direction of DE was not concordant between TRM cells and TGF-β-treated groups. The numbers in the pie chart denote the percentage overlap between the genes in the TRM-related signature and genes DE (FDR < 0.05) in the TGF-β-treated groups. The *P*-values next to the pie charts denote the enrichment *P*-values calculated via bootstrapping (Methods).

Given that TGF-β has been shown to induce CD103 expression in the skin, lung, and gut [8,11,12,17,18], we hypothesized that the residency-related TRM cell genes shared across these tissues might reflect further TGF-β imprinting. We therefore sought to determine whether TGF-β stimulated CD8^+^ T cells established under *in vitro* conditions share a gene signature with murine TRM cells isolated from these three tissues. To do so, the list of DE genes in each of the TGF-β-treated groups was compared against genes previously established as the core TRM-related signature [12]. The core TRM-related signature consisted of 35 genes that were significantly up- or down- regulated in murine TRM cells from skin, gut, and lung in comparison to their circulating spleen counterparts. We found 46–60% of these genes were consistently expressed in a similar manner in our TGF-β-treated groups (**Fig 4)**. The overlap between the TRM core signature and the full set of differentially expressed genes in all the three treatment comparisons was statistically significant (*P* < 0.001) in a bootstrapping permutation test (**Methods**). The majority of the overlapping TRM core signature genes were up-regulated in the TGF-β-treated groups, consistent with the hypothesis that TGF-β induces genes that promote the maintenance of TRM cells in tissues. The TRM core genes that were consistently up-regulated in all the TGF-β-treated groups included *Cdh244*, *Chd1*, *Chn2*, *Hpgds*, *Inpp4b*, *Itga1*, *Itgae*, *Qpct*, *Rgs1*, *Rgs2* and *Skil*. *Fam65b* was the only TRM-related gene that was consistently down-regulated across all the TGF-β-treated groups.

**Fig 5:**
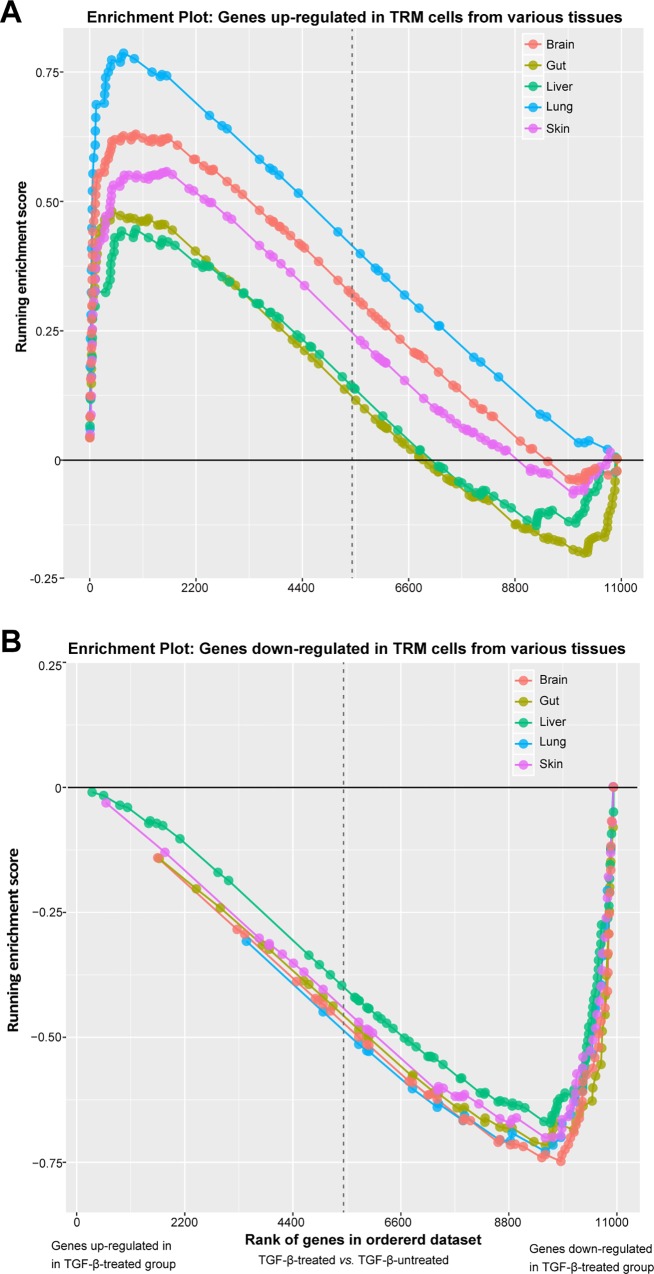
Enrichment plot for tissue-specific TRM-associated gene sets in the TGF-β-treated group. GSEA enrichment plots for the **(A)** five TRM-related up-regulated and **(B)** five TRM-related down-regulated gene sets in the TGF-β-treated group. All 10 gene sets shown were significantly enriched at *P*-value < 0.05 and FDR q-value < 0.25. Each plot shows the running enrichment scores (y-axes) and the position (denoted by dots) of the members of each gene set in the ranked list of genes DE between TGF-β-treated group and TGF-β-untreated group (x-axes). The genes in the ranked list are ordered along the x-axes based on fold change, where the most up-regulated genes in the TGF-β-treated group are on the far left and the most down-regulated genes on the far right. The dotted vertical grey line represents fold change of zero. The curved lines, coloured by tissue type, show the cumulative enrichment score.

Next, we examined if TGF-β influenced the transcriptional signature of TRM cells in a tissue-specific manner. To do so, we performed GSEA to test whether the differentially expressed genes in the TGF-β-treated groups were enriched for tissue-specific TRM cell-associated genes from both epithelial (skin, gut, and lungs) and non-epithelial tissues (brain and liver). The tissue-specific TRM cell-associated gene sets comprised genes previously identified as either up- or down-regulated in murine TRM cells from skin, gut, lung, brain, and liver in comparison to their circulating counterparts [12,31,32]. The list of all 10 up- and down-regulated gene sets is provided in **Table A in S1 Table** (see Methods for details). GSEA confirmed that all five up-regulated and five down-regulated gene sets were significantly (*P*-value < 0.05) enriched in the DE genes in our TGF-β-treated groups (**Fig 5 and Table D in S1 Table**). Surprisingly, despite the absence of CD103 expression on liver TRM cells, we found that liver TRM gene sets were also significantly enriched. Genes that were up-regulated in TRM cells had higher expression in the TGF-β-treated groups (**Fig 5A**), whereas down-regulated genes had higher expression in the TGF-β-untreated groups (**Fig 5B**). By contrast, TEM- and TCM-related gene sets (negative control gene sets) showed no enrichment for the TGF-β-responsive transcriptional profile (**S4 Fig and Table D in S1 Table)**. Taken together, these results support the role of TGF-β signalling in shaping the transcriptional signature in TRM cells across multiple diverse tissues. This further suggests that TGF-β has a broad impact on the transcriptional machinery underlying tissue residency in different TRM cell populations.

## Discussion

TRM cells play an important role in providing first-line defence at barrier sites [2,3,5–9]. However, the tissue-specific signals required for their differentiation and long-term persistence at these sites are not well established. An improved understanding of how local tissue factors contribute to the generation and maintenance of TRM cells will be important for the development of vaccines and immunotherapies designed to elicit tissue-directed immunity. TGF-β is by far the best characterised tissue-derived signal known to be required for the differentiation of CD103^+^ TRM cells in the skin, gut, and lungs by inducing CD103 expression [8,11,12,17,18]. Here we show that TGF-β-stimulated CD8^+^ T cells established under *in vitro* conditions acquired a gene expression signature enriched for TRM-related genes from multiple tissues.

The comparable 40–60% expression pattern similarity expands our view of TGF-β as a factor required for the initial generation of TRM cells to a more broadly acting cytokine influencing the majority of TRM-associated genes, which are most likely linked to their survival and persistence. Our results are consistent with previous studies showing that continuous signalling by TGF-β is required for TRM cells to be locally maintained long-term [33,34]. Our analysis further revealed that the TGF-β induced signature was significantly enriched for TRM-related genes identified in TRM cells from both epithelial (skin, gut, and lung) and non-epithelial (brain and liver) tissues. This suggests that TGF-β signalling might play an important role in shaping the common transcriptional program that underpins tissue residency of TRM cell populations across organs.

Several TRM-associated genes that play an essential role in tissue residency were common in the TGF-β induced gene signature. *Itgae*, which encodes for CD103, was one of the most significantly up-regulated genes in the TGF-β-treated group, consistent with previous findings that TGF-β induces CD103 expression on TRM cells [11,18,35]. CD103 binds to its ligand E-cadherin, which is expressed on the epithelial surfaces of the skin and gut; possibly meditating the retention of TRM cells by tethering them within these tissues [36,37]. It has been shown that CD103-deficient mice had lower T cell numbers in skin, intestinal, and vaginal epithelium [12,36,38] in the memory phase, further suggesting that TGF-β induced expression of CD103 is important for tissue retention of TRM cells. Moreover, the long-term retention of CD103 deficient T cells was not as severely impaired compared to cells lacking the TGF-β receptor [17]. Hence, the effect of TGF-β signalling on TRM cells must be broader than just for the initial induction of CD103 and subsequent adherence of TRM cells. Additionally, *Cdh1* and *Itga1*, which also encode for adhesion molecules, were up-regulated in the TGF-β-treated group. *Cdh1* and *Itga1* genes encode for the E-cadherin and alpha 1 subunit of integrin receptors, respectively, and have been previously reported to be up-regulated in TRM cells [12,31,39]. In Langerhans cells, TGF-β dependent induction of E-cadherin is crucial for their residency and maintenance in the skin [40], implicating a similar requisite in TRM cells. Indeed, genetic deficiency in E-cadherin expression has been shown to result in a defect of CD103^+^ TRM generation in salivary glands [14]. Increased expression of the chemokine *Xcl1* was also noted in the TGF-β-treated group. Similarly, several studies have reported high expression levels of *Xcl1* in TRM cells [12,31,39]. We also observed several other genes previously found to be highly expressed in TRM cells to be consistently up-regulated in the TGF-β-treated group. This included genes encoding for costimulatory receptors involved in immunomodulation (*Ctla4*, and *Icos)*, enzymes *(Inapp4b* and *Qpct)*, and signalling regulators that mediate tissue retention (*Rgs1 and Rgs2*) [12,31,39]. Hence, the significant overlap seen between genes involved in TRM cell retention and those in the TGF-β induced signature suggests that TGF-β dependent induction of these genes may be broadly important for the establishment of residency by TRM cells at tissue sites.

A potential limitation of this study is that it used *in vitro* stimulated CD8^+^ T cells as a surrogate for the differentiation of TRM cells. The development and tissue-specific activation of TRM cells likely require precise temporal and spatial regulation of gene expression, which may be achieved by a combination of locally expressed cytokines and by epigenetic mechanisms such as histone modifications and DNA methylation. Hence, it is likely that epigenetic changes and/or tissue-specific local cues other than TGF-β will have an additional impact on the transcriptional signature of TRM cells at tissue sites *in vivo*. However, the *in vitro* activated cells treated with TGF-β appear very similar to TRM cells, since more than half of the genes were regulated in a way that is reminiscent of the regulation of genes observed in TRM versus circulating cells in previous studies. Thus, our analyses corroborate the central role TGF-β plays in imprinting a transcriptional profile in TRM cells. Nevertheless, further *in vivo* studies are required to establish how constant “education” by TGF-β directs TRM cells to acquire long-term maintenance capacity in various epithelial and non-epithelial tissue sites.

## Supporting information

S1 Fig Analysis pipeline for RNA-seq data. Quality control of raw PE reads (FASTQ format) was performed using FastQC. Bowtie2 and Tophat2 were used to align the raw reads to the mm10 version of the mouse reference genome (downloaded from the UCSC browser). Mapped reads from BAM files together with a reference gene annotation file (GTF format) were supplied to Cufflinks2 for transcript assembly and quantification. Differential analysis was performed using Cuffdiff2. Three pairwise comparisons were made: (1) TGF-β *vs*. Untreated; (2) IL-2/TGF-β *vs*. Untreated; (3) IL-2/TGF-β *vs*. IL-2.
(TIF)

S2 Fig Heatmap from the hierarchical clustering of top 30 most differentially expressed genes (FDR < 0.05, |log2FC| > 2) common in all three comparisons. The FPKM expression values for each gene across the 12 samples are presented after being log2 transformed and scaled (mean of 0 and standard deviation of 1), such that red denotes increased expression and blue denotes decreased expression. The dendrogram shows the clustering of the samples based on the expression of the 30 genes and the branches are coloured blue for TGF-β-treated groups and orange for TGF-β-untreated groups. Circles represent the samples, which are coloured according to the treatment they received, and the numbers inside denote each biological replicate.
(TIF)

S3 Fig Flow cytometric analysis of activated gBT-I cells after 4 days of activation and 40h of TGF-β treatment. After pre-gating on live singlets and vα2+ cells, staining of CD103 (Itgae protein). The histogram is representative of over 3 independent experiments.
(TIF)

S4 Fig TCM and TEM-related genes from GSEA. Enrichment plots for the (A) TCM and TEM-related up-regulated gene sets and (B) TEM-related down-regulated gene set in the TGF-β-treated group. None of the gene sets were significantly enriched at *P*-value < 0.05 and FDR *q*-value < 0.25. Each plot shows the running enrichment scores (y-axis) and the position of the members in each gene set in the ranked list of genes DE between TGF-β-treated-group and TGF-β-untreated group (x-axis). The genes in the rank list are ordered along the x-axis based on fold change, where the most up-regulated genes in the TGF-β-treated group are on the far left and the most down-regulated genes on the far right. The dotted vertical grey line represents fold change of zero. The curved lines, colored by TCM or TEM, show the cumulative enrichment score. The dots denote the positions in the ordered ranked list where the genes in each gene set appear. Of note, the TCM-related downregulated gene set was not tested for enrichment since it did not achieve the minimum gene set size of N = 15.
(TIF)

S1 Table (XLSX)
