# Supplementary figures and images for: Comparative analysis reveals a role for TGF-β in shaping the residency-related transcriptional signature in tissue-resident memory CD8+ T cells
Source: PLoS One. 2019 Feb 11;14(2):e0210495. doi: 10.1371/journal.pone.0210495 (PMC6370189; doi:10.1371/journal.pone.0210495)

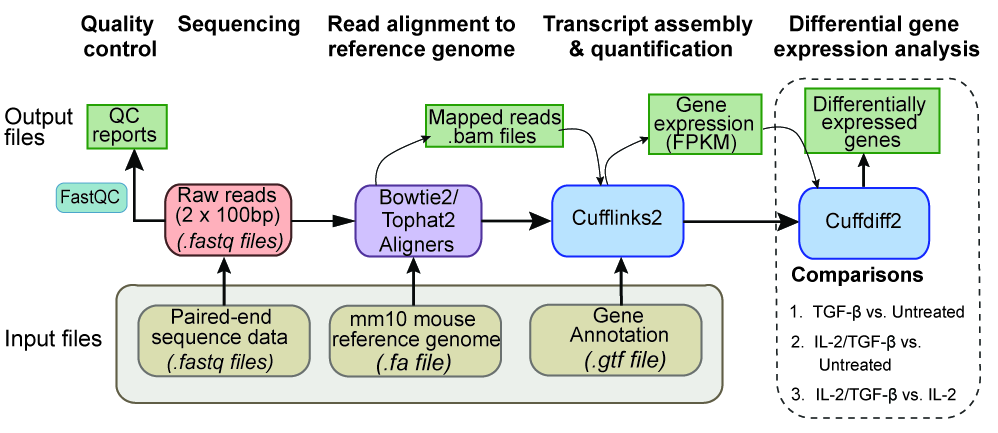

Supplement: S1 Fig — Quality control of raw PE reads (FASTQ format) was performed using FastQC. Bowtie2 and Tophat2 were used to align the raw reads to the mm10 version of the mouse reference genome (downloaded from the UCSC browser). Mapped reads from BAM files together with a reference gene annotation file (GTF format) were supplied to Cufflinks2 for transcript assembly and quantification. Differential analysis was performed using Cuffdiff2. Three pairwise comparisons were made: (1) TGF-β vs. Untreated; (2) IL-2/TGF-β vs. Untreated; (3) IL-2/TGF-β vs. IL-2. (TIF) [file pone.0210495.s001.tif]

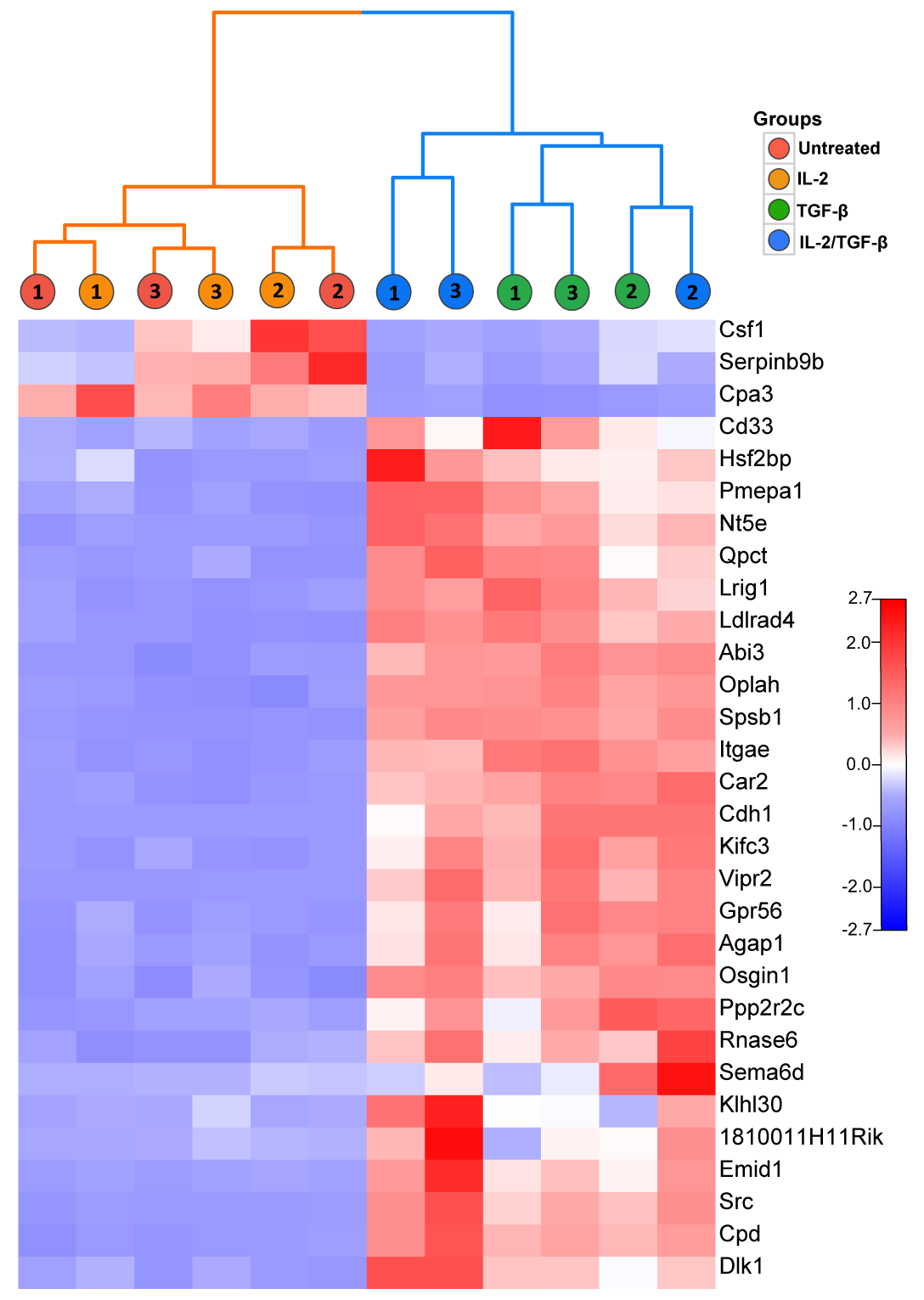

Supplement: S2 Fig — The FPKM expression values for each gene across the 12 samples are presented after being log2 transformed and scaled (mean of 0 and standard deviation of 1), such that red denotes increased expression and blue denotes decreased expression. The dendrogram shows the clustering of the samples based on the expression of the 30 genes and the branches are coloured blue for TGF-β-treated groups and orange for TGF-β-untreated groups. Circles represent the samples, which are coloured according to the treatment they received, and the numbers inside denote each biological replicate. (TIF) [file pone.0210495.s002.tif]

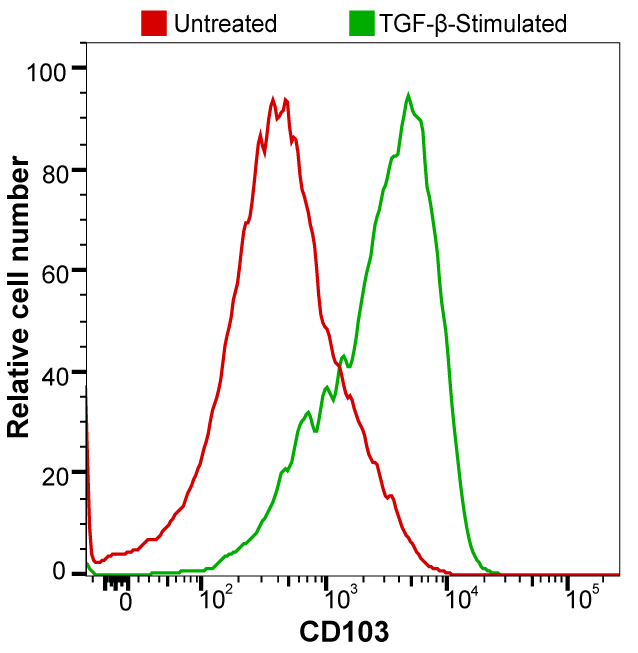

Supplement: S3 Fig — After pre-gating on live singlets and vα2+ cells, staining of CD103 (Itgae protein). The histogram is representative of over 3 independent experiments. (TIF) [file pone.0210495.s003.tif]

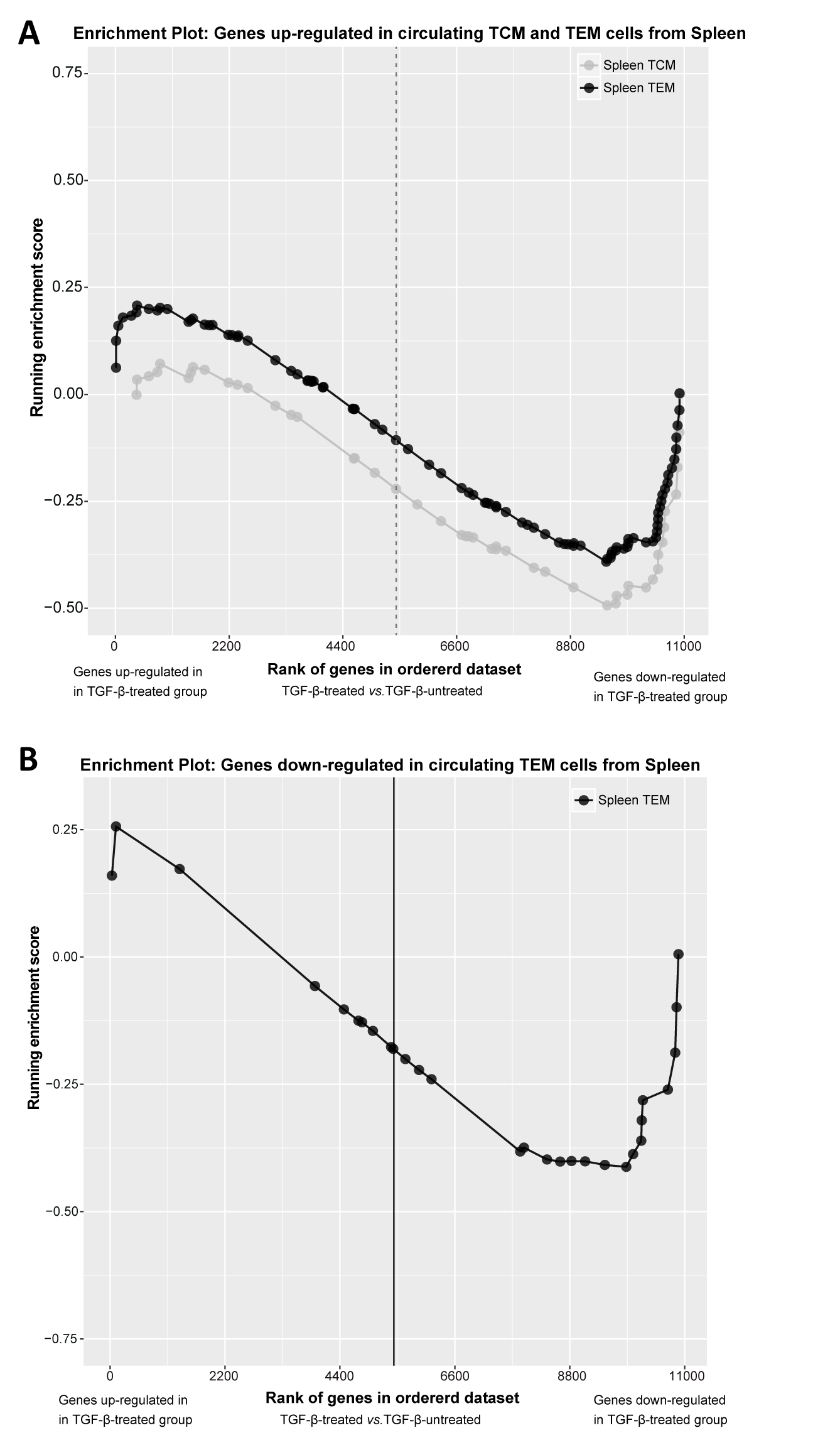

Supplement: S4 Fig — Enrichment plots for the (A) TCM and TEM-related up-regulated gene sets and (B) TEM-related down-regulated gene set in the TGF-β-treated group. None of the gene sets were significantly enriched at P-value < 0.05 and FDR q-value < 0.25. Each plot shows the running enrichment scores (y-axis) and the position of the members in each gene set in the ranked list of genes DE between TGF-β-treated-group and TGF-β-untreated group (x-axis). The genes in the rank list are ordered along the x-axis based on fold change, where the most up-regulated genes in the TGF-β-treated group are on the far left and the most down-regulated genes on the far right. The dotted vertical grey line represents fold change of zero. The curved lines, colored by TCM or TEM, show the cumulative enrichment score. The dots denote the positions in the ordered ranked list where the genes in each gene set appear. Of note, the TCM-related downregulated gene set was not tested for enrichment since it did not achieve the minimum gene set size of N = 15. (TIF) [file pone.0210495.s004.tif]
